# Development of an integrated clinical-laboratory scoring system for accurate HIT diagnosis

**DOI:** 10.1182/bloodadvances.2025018867

**Published:** 2026-02-10

**Authors:** Günalp Uzun, Sergio Origel Romero, Jan Zlamal, Johann Jacoby, Oliver Borst, Peter Rosenberger, Sven Poli, Stefanie Hammer, Tamam Bakchoul, Karina Althaus

**Affiliations:** 1 Center for Clinical Transfusion Medicine, University of Tuebingen, Tuebingen, Germany; 2 Institute for Clinical and Experimental Transfusion Medicine, University of Tuebingen, Tuebingen, Germany; 3 Institute of Clinical Epidemiology and Applied Biometry, University Hospital Tuebingen, University of Tuebingen, Tuebingen, Germany; 4 Department of Cardiology and Angiology, University Hospital Tuebingen, Tuebingen, Germany; 5 DFG Heisenberg Group Cardiovascular Thrombo-Inflammation and Translational Thrombocardiology, University of Tuebingen, Tuebingen, Germany; 6 Department of Anaesthesiology and Intensive Care Medicine, University Hospital Tuebingen, Tuebingen, Germany; 7 Department of Neurology and Stroke, University Hospital Tuebingen, Tuebingen, Germany; 8 Hertie Institute for Clinical Brain Research, University of Tuebingen, Tuebingen, Germany

**TO THE EDITOR:**

Heparin-induced thrombocytopenia (HIT) is a severe complication of heparin therapy, caused by antibodies against platelet factor 4/heparin complexes. Rapid and reliable diagnosis is essential, because delayed recognition increases thrombotic risk, whereas overdiagnosis may lead to overtreatment and bleeding complications.

The American Society of Hematology (ASH) recommends a diagnostic approach including the 4Ts score, laboratory detection of platelet factor 4/heparin antibodies with immunoassays (IA), and confirmatory functional testing (supplemental Figure 1).^1^ However, in practice, the 4Ts score lacks specificity,^2^^,^^3^ whereas IA are frequently false positive.^4^, ^5^, ^6^, ^7^, ^8^, ^9^ Therefore, functional assays, such as the heparin-induced platelet activation (HIPA) assay^10^ or the serotonin-release assay,^11^ remain the diagnostic gold standard. The drawbacks of these assays are that they are technically demanding, restricted to specialized laboratories, and rarely deliver results within the timeframe needed for urgent treatment decisions. Recent approaches that combine IA with clinical or statistical algorithms include the “Lausanne” algorithms (sequential chemiluminescence IA [CLIA] and latex immunoturbidimetric assay [LIA] with likelihood ratios),^12^ the “Hamilton” algorithm (6-point scoring system using simultaneous CLIA and LIA),^13^ and “TORADI-HIT” (machine learning integration of clinical variables with IA results).^14^ However, these algorithms remain center specific, require either dual IA platforms or web-based calculators, and have not yet achieved widespread clinical adoption.

In this single-center study, we developed a multivariable prediction model for the diagnosis of HIT in hospitalized patients with suspected disease. The Tuebingen HIT (TuHIT) score integrates clinical and laboratory parameters into a single additive score. We also compared its diagnostic performance with 2- and 3-stage diagnostic strategies recommended by the ASH guidelines.^1^

The study cohort consisted of 400 consecutive adult patients with suspected HIT between 1 October 2023 and 28 February 2025 at the University Hospital of Tuebingen. The 4Ts scores were calculated by treating physicians at the time of sample submission. All patient samples were analyzed by CLIA (HemosIL AcuStar HIT immunoglobulin G [IgG]; Werfen, München, Germany), IgG-specific enzyme-linked immunosorbent assay (ELISA; Zymutest HIA IgG; Hyphen BioMed, Neuville-sur-Oise, France), and the in-house HIPA assay. HIT diagnosis was based on HIPA results and clinical data. The 4Ts scores were categorized according to pretest probability, as defined by ASH guidelines: low (≤3 points), intermediate (4-5 points), and high (6-8 points).^1^ CLIA and ELISA results were stratified using investigator-defined or manufacturer-recommended cutoffs (Figure 1; supplemental Table 1). Statistical optimization of the point allocation and cutoffs was performed using logistic regression modeling and receiver operating characteristic (ROC) curve analysis (supplemental Table 2). Points were assigned to each component and then summed to yield the TuHIT score for each patient. Detailed methods are provided in the supplemental Methods. The study was conducted in accordance with the declaration of Helsinki. The study protocol was approved by the Ethics Committee at the Medical Faculty of the University of Tuebingen.

**Figure 1.**
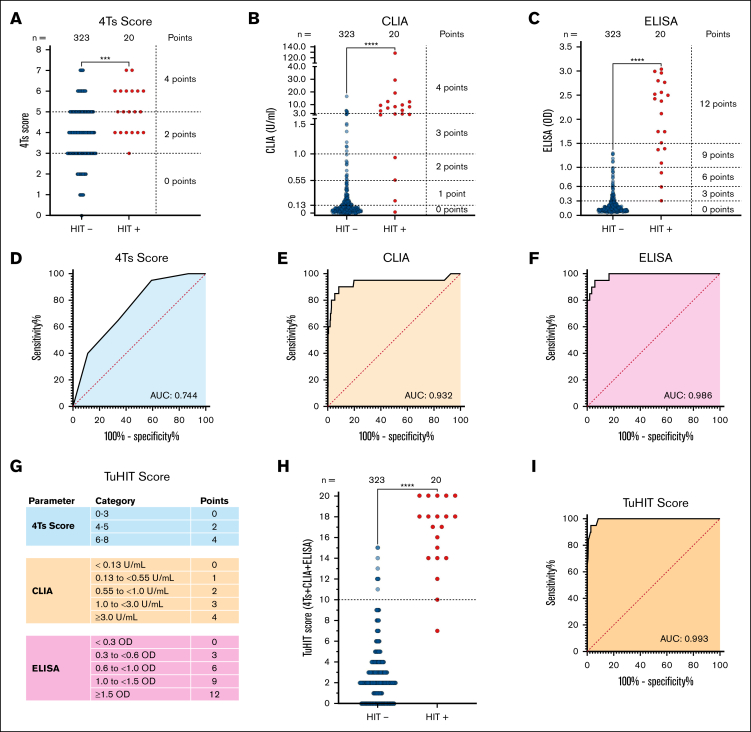
**Diagnostic performance of 4Ts score, IA, and the TuHIT score.** Distribution of 4Ts score (A), CLIA values (units per milliliter) (B), and ELISA OD (C) in patients with (HIT^+^; red) and without HIT (HIT^−^; blue). Each dot represents 1 patient. The horizontal dashed lines indicate cutoff thresholds for categorical point assignment. Statistical comparisons were performed using the Mann-Whitney *U* test for continuous variables and Fisher exact test for categorical distributions (∗∗∗*P* < .001; ∗∗∗∗*P* < .0001). ROC curves for 4Ts score (AUC, 0.744; 95% confidence interval [CI], 0.648-0.841; *P* = .0002) (D), CLIA (AUC, 0.932; 95% CI 0.845-1.00; *P* < .0001) (E), and ELISA (AUC, 0.986; 95% CI 0.969-1.00; *P* < .0001) (F). The shaded areas depict sensitivity vs 1 − specificity, with the corresponding AUC values indicated. (G) TuHIT score showing point allocation: 4Ts (0-4 points; blue), CLIA (0-4 points; orange), and ELISA (0-12 points; purple), total range 0 to 20 points. Example of TuHIT score calculation is as follows: a patient with a 4Ts score of 5 (2 points), CLIA of 2.5 U/mL (3 points), and ELISA of 1.2 OD (9 points) has a TuHIT score of 14 points (2 + 3 + 9 = 14). (H) TuHIT score distribution in patients who were HIT^−^ and HIT^+^. The dashed line indicates optimal diagnostic threshold (≥10 points; determined by Youden index). (I) ROC curve for TuHIT score (AUC, 0.993; 95% CI 0.984-1.000; *P* < .0001).

After excluding 57 repeat samples, 343 patients were included (supplemental Figure 2). The cohort comprised 215 males (62.7%) and 128 females (37.3%), with a median age of 65 years (interquartile range, 54-74). There were no missing data for the main parameters (4Ts, CLIA, ELISA, and HIPA). Twenty patients (5.8%) were diagnosed with HIT (supplemental Table 3).

When evaluated individually by ROC analysis, the 4Ts score showed limited discrimination between patients with HIT and without HIT (area under the ROC curve [AUC], 0.744; *P* = .0002; Figure 1A,D). A cutoff ≥4 resulted in high sensitivity but poor specificity (Table 1). CLIA at the manufacturer's threshold (1.0 U/mL) showed 80.0% sensitivity and 95.0% specificity; a ROC-guided threshold (0.55 U/mL; AUC, 0.932; *P* < .0001; Figure 1B,E) improved sensitivity to 90.0% while maintaining 91.6% specificity (Table 1). ELISA at optical density (OD) ≥0.30 achieved 100.0% sensitivity with 82.7% specificity; at the ROC-guided threshold (OD, ≥0.60; AUC, 0.986; *P* < .0001; Figure 1C,F), specificity increased to 94.1% with 90.0% sensitivity (Table 1). Across assays, negative predictive values (NPVs) exceeded 98.7%, supporting IA use to rule out HIT.

**Table 1.** **Diagnostic performance of single assays, 2-stage and 3-stage ASH diagnostic strategies, and the TuHIT score**

Diagnostic strategy	Positive, n (%)		Negative, n (%)		% (95% confidence interval)				
	True	False	True	False	Sensitivity	Specificity	PPV	NPV	Accuracy
**Single assay**									
4Ts score ≥4	19 (95.0)	191 (59.2)	132 (40.8)	1 (20.0)	95.0 (75.1-99.8)	40.9 (35.5- 46.5)	9.0 (8.0-10.2)	99.2 (95.1-99.9)	44.0 (38.7-49.5)
CLIA alone >1.0 U/mL	16 (80.0)	16 (5.0)	307 (95.0)	4 (20.0)	80.0 (56.3-94.3)	95.0 (92.1-96.7)	50.0 (37.2-62.8)	98.7 (96.7-99.5)	94.2 (91.1-96.4)
CLIA alone ≥0.55 U/mL	18 (90.0)	27 (8.4)	296 (91.6)	2 (10.0)	90.0 (68.3-98.8)	91.6 (88.1-94.4)	40.0 (31.1-49.6)	99.3 (97.5-99.8)	91.6 (88.1-94.3)
ELISA alone ≥0.3 OD	20 (100.0)	56 (17.3)	267 (82.7)	0 (0.0)	100.0 (83.2-100.0)	82.7 (78.1-86.6)	26.3 (21.9-31.2)	100.0 (98.6-100.0)	83.7 (89.4-87.4)
ELISA alone ≥0.6 OD	18 (90.0)	19 (5.9)	304 (94.1)	2 (10.0)	90.0 (68.3-98.8)	94.1 (91.0-96.4)	48.6 (37.4-60.0)	99.4 (97.6-99.8)	93.9 (90.8-96.2)
**2-Stage ASH strategy (4Ts score + CLIA or ELISA)**									
4Ts + CLIA >1.0 U/mL	16 (80.0)	11 (3.4)	312 (96.6)	4 (20.0)	80.0 (56.3-94.3)	96.6 (93.4-98.3)	59.3 (43.4-73.0)	98.7 (97.0-99.5)	95.6 (92.9-97.5)
4Ts + CLIA >0.55 U/mL	17 (85.0)	18 (5.5)	305 (94.5)	3 (15.0)	85.0 (62.1-96.8)	94.4 (92.3-96.7)	48.6 (36.8-60.5)	99.0 (97.3-99.7)	93.9 (90.8-96.2)
4Ts + ELISA >0.3 OD	19 (95.0)	37 (10.7)	286 (89.3)	1 (5.0)	95.0 (75.1-99.9)	88.5 (84.6-91.2)	33.9 (27-2-41.4)	99.7 (97.7-100.0)	88.9 (85.1-92.0)
4Ts + ELISA >0.6 OD	17 (85.0)	15 (4.6)	308 (95.4)	3 (15.0)	85 (62.1-96.8)	95.4 (92.5-97.4)	53.1 (40.1-65.8)	99.0 (97.3-99.7)	94.8 (91.8-96.9)
4Ts + ELISA >1.0 OD	16 (80.0)	6 (1.8)	317 (98.2)	4 (20.0)	80.0 (56.3-94.3)	98.1 (96-99.3)	72.7 (54.0-85.9)	98.8 (97.1-99.5)	97.1 (94.7-98.6)
4Ts + ELISA >1.5 OD	13 (65.0)	0 (0.0)	323 (100.0)	7 (35.0)	65.0 (40.8-84.6)	100.0 (98.7-100.0)	100.0 (75.3-100.0)	97.9 (96.2-98.8)	98.0 (95.8-99.2)
**3-Stage ASH strategy with functional assay (4Ts score + CLIA or ELISA + HIPA)**									
4Ts + CLIA >1.0 U/mL + HIPA	16 (80.0)	0 (0.0)	323 (100.0)	4 (20.0)	80.0 (56.3-94.3)	100.0 (98.9-100.0)	100.0 (79.4-100.0)	98.8 (97.1-99.5)	98.8 (97.0-99.7)
4Ts + CLIA >0.55 U/mL + HIPA	17 (85)	0 (0.0)	323 (100.0)	3 (15)	85.00 (62.1-96.8)	100.0 (98.9-100.0)	100.0 (80.5-100.0)	99.1 (97.4-99.7)	99.1 (97.5-99.8)
4Ts + ELISA ≥0.3 OD + HIPA	18 (90.0)	0 (0.0)	323 (100.0)	2 (10.0)	90.0 (68.3-98.8)	100.0 (98.9-100.0)	100.0 (81.5-100.0)	99.4 (97.8-99.8)	99.4 (98.0-99.9)
4Ts + ELISA ≥0.6 OD + HIPA	16 (80.0)	0 (0.0)	323 (100.0)	4 (20.0)	80.0 (56.3-94.3)	100.0 (98.9-100.0)	100.0 (79.4-100.0)	98.8 (97.1-99.5)	98.8 (97.0-99.7)
TuHIT score ≥10	19 (95.0)	9 (2.7)	314 (97.3)	1 (5.0)	95.0 (75.1-99.9)	97.2 (94.8-98.7)	67.9 (52.4-80.2)	99.7 (97.9-100.0)	97.1 (94.7-98.6)

Coupling the 4Ts score with IA in 2-stage ASH diagnostic strategies improved overall accuracy but left sensitivity gaps and modest positive predictive values (PPVs). Using 4Ts combined with CLIA >1.0 U/mL yielded 80.0% sensitivity, high specificity, and NPV, but modest PPV (Table 1). Using 4Ts combined with ELISA ≥0.30 achieved the highest sensitivity (95.0%) but with lower specificity and PPV. Increasing the threshold to ≥0.60 OD provided more balanced performance with 85.0% sensitivity and improved PPV, while maintaining high NPV and accuracy (Table 1). Higher thresholds (1.0-1.5 OD) progressively reduced false positives but also decreased sensitivity (Table 1). Depending on IA choice and cutoff, 2-stage pathways would have missed 5% to 35% of HIT patients while generating many false positives requiring further clarification.

Adding functional testing in 3-stage ASH strategies eliminated false positives (100% specificity and PPV) and increased accuracy to 98.8% to 99.4% (Table 1). However, sensitivity remained constrained by IA thresholds at 80% to 90%, meaning up to 20% of HIT patients could be missed with IA-based exclusion. For resource planning, 3-stage pathways would have triggered functional assays in 27 to 56 patients (8%-16% of the cohort).

In contrast, the TuHIT score exhibited superior discrimination (AUC, 0.993; 95% confidence interval, 0.984-1.000; *P* < .0001; Figure 1G-I) with a better balance between sensitivity and specificity. At a cutoff ≥10 points, TuHIT achieved 95% sensitivity, 97.2% specificity, and 97.1% overall accuracy (Table 1).

These performance metrics compare favorably with other published combined IA algorithms. The “TORADI-HIT” algorithm achieved 89% to 100% sensitivity depending on the IA used, but with modest PPV of 61% to 62% in the original study^,^^14^ and showed a 6.3% false-negative rate and could only be applied to half of patients due to missing data in the external validation cohort.^12^ The “Hamilton” algorithm reported high sensitivity (99%) with simultaneous dual IA testing but produces 10% unsolved cases.^12^^,^^13^ The “Lausanne” sequential approach (CLIA→LIA or LIA→CLIA) achieved NPV and PPV over 95% with 0 false negatives and required a second IA in <20% of patients.^12^ Although highly accurate, Bayesian approaches require probability tables and may be less practical for routine clinical use. In comparison, TuHIT provides comparable diagnostic accuracy through a single standardized point-based score, potentially facilitating broader clinical implementation.

Three main implications emerge from these data. First, single IA already provide strong rule out, as reflected by consistently high NPVs, but relying on the 4Ts score alone to triage the testing is unsatisfactory, given its low specificity and high rate of false-positive results. Second, 2-stage ASH strategies (4Ts + IA) improve the specificity compared with IA alone, yet still miss a relevant proportion of HIT patients and frequently generate positive results requiring functional confirmation. Third, the TuHIT score, by integrating clinical probability with quantitative IA results, achieves both excellent sensitivity and high specificity, thereby reducing the need for functional assay and offering a pragmatic alternative for centers without immediate access to these assays.

Strengths of our study include enrollment of consecutive real-world referrals, head-to-head testing with both IA and a reference functional assay, and a clinically diverse population enhancing generalizability. Furthermore, the TuHIT score is straightforward, requiring no specialized software or training.

Limitations include the small number of HIT-positive patients yielding wide CIs and limiting statistical power. External validation in independent cohorts is needed to confirm generalizability. The single-center design may not reflect practice patterns or patient populations elsewhere. Implementation requires clinicians familiar with HIT diagnostic criteria and laboratories capable of performing both CLIA and ELISA; if only 1 IA is available, standard diagnostic algorithms should be used.

In conclusion, we developed a point-based integrative scoring system for HIT diagnosis. Its accuracy is comparable to other recently proposed diagnostic approaches, but with the practical advantage of providing a single, standardized numerical score. TuHIT may serve as a pragmatic tool to facilitate evidence-based decision-making in suspected HIT patients, especially in settings where rapid access to functional assays is limited. However, external validation through a multicenter study is essential to confirm the reliability and generalizability of this diagnostic approach before it can be recommended for routine clinical use.

**Conflict-of-interest disclosure:** K.A. reports honoraria to the Medical University Hospital of Tuebingen for lectures from CSL Behring, Biotest, Sobi, Meet The Experts, Expanda, and Werfen; and research grants to the Medical University Hospital of Tuebingen from Octapharma, Takeda, and Bayer, unrelated to this work. T.B. reports research funding from CoaChrom Diagnostica GmbH, Deutsche Forschungsgemeinschaft, Robert Bosch GmbH, Stiftung Transfusionsmedizin und Immunhämatologie e.V., Ergomed, DRK Blutspendedienst, Deutsche Herzstiftung, Ministerium fuer Wissenschaft, and Forschung und Kunst Baden-Wuerttemberg; lecture honoraria from Meet The Experts Academy UG, Schoechl Medical Education GmbH, Novo Nordisk Pharma GmbH, and Swedish Orphan Biovitrum GmbH; has provided consulting services to Terumo; and has provided expert witness testimony relating to heparin-induced thrombocytopenia (HIT) and non-HIT thrombocytopenic and coagulopathic disorders (unrelated to this work). The remaining authors declare no competing financial interests.

## Supplementary Material

Supplemental Methods, Figures, Tables, and References

Supplemental Data (TRIPOD-AI Checklist)
