# Supplementary material for: Development of an integrated clinical-laboratory scoring system for accurate HIT diagnosis
Source: Blood Adv. 2026 Feb 10;10(8):2829–32. doi: 10.1182/bloodadvances.2025018867 (PMC13122791; doi:10.1182/bloodadvances.2025018867)
Supplement: Supplemental Methods, Figures, Tables, and References [file BLOODA_ADV-2025-018867-mmc1.pdf]

Supplemental Material

Development of an integrated clinical–laboratory scoring system for accurate HIT diagnosis

Günalp Uzun[1,2], Sergio Origel Romero[1], Jan Zlamal[1,2], Johann Jacoby[3], Oliver Borst[4,5], Peter Rosenberger[6], Sven Poli[7,8], Stefanie Hammer[1,2], Tamam Bakchoul[1,2], Karina Althaus[1,2]

Index

Methods 2
Study cohort 2
Immunoassays 3
Heparin induced platelet activation (HIPA) assay 3
Diagnostic algorithm for HIT diagnosis 3
ASH recommended diagnostic algorithm for HIT diagnosis 5
Development of the TuHIT score 5
Statistics 8
Results 9
References 12

Methods

Study cohort

This was a single-center study conducted at the University Hospital of Tübingen, a tertiary care academic medical center in Germany. Study cohort consisted of all hospitalized adult patients (≥18 years) referred to our diagnostic laboratory with clinical suspicion of HIT between 01.10.2023 and 28.02.2025. Diagnostic suspicion was determined by treating physicians based on clinical assessment, and referral was defined as any request for laboratory investigation for HIT. Patients were eligible regardless of underlying disease, clinical specialty (medical, surgical, or intensive care), or treatment modality. Patients were excluded from the final analysis if they had (1) repeat testing during the same hospitalization (only the first sample was included), or (2) incomplete clinical or laboratory data essential for TuHIT score calculation.

Clinical data, including demographic information, were extracted from the hospital's electronic medical record system. Laboratory values were obtained from the department information system. The 4Ts scores were initially calculated by treating physicians at the time of sample submission using standardized forms. During subsequent data collection and analysis, all 4Ts scores were independently verified and recalculated by two experienced physicians based on comprehensive review of clinical patient records to ensure accuracy and consistency.

All patients received standard medical care. Treatment decisions (including anticoagulation management) were made by treating physicians and did not influence model development.

Multiple strategies were implemented to ensure data quality and integrity during retrospective data collection. Data were extracted systematically from electronic medical records and laboratory information systems. Two independent reviewers (S.O.R and K.A.) performed data abstraction for key variables, including 4Ts score components, immunoassay results, and HIPA outcomes, with discrepancies resolved through consensus review. Range checks were applied to all continuous variables (e.g., platelet counts, laboratory values) to flag physiologically implausible values for manual verification against source documents. Consistency checks were performed between related variables to identify potential extraction errors or logical inconsistencies (e.g., timing of thrombocytopenia relative to heparin exposure, platelet nadir values). All main parameters essential for TuHIT score calculation (4Ts score components, CLIA results, ELISA results, and HIPA outcomes) were complete with no missing data. The conversion of clinical and laboratory parameters into TuHIT score points using predefined cut-offs was applied consistently across all patient subgroups.

Immunoassays

We employed HemosIL AcuStar® HIT-IgG (Werfen, Barcelona, Spain), a fully automated CLIA, and ZYMUTEST HIA IgG (Hyphen BioMed, Neuville-sur-Oise, France), a microplate-based ELISA, to determine Anti-PF4/heparin IgG antibodies.

HemosIL AcuStar® HIT-IgG employs paramagnetic microparticles coated with PF4/polyanion complexes to capture patient IgG antibodies. Bound antibodies are detected by an isoluminol-labeled anti-human IgG conjugate, and the resulting chemiluminescent signal is measured by the AcuStar analyzer within 30 minutes. Results were expressed as arbitrary units (U/mL), with values ≥1.0 U/mL considered positive according to the manufacturer's instructions.

In ZYMUTEST HIA IgG, two hundred microliters of diluted patient sample (1:100) and 50 µL of platelet lysate containing PF4 were added to empty wells of a microtiter plate coated with protamine sulfate and unfractionated heparin. Bound antibodies are detected with a horseradish peroxidase–conjugated anti-human IgG antibody, followed by colorimetric measurement at 450 nm using a microplate reader. Optical density (OD) values were expressed in arbitrary units (AU). An OD < 0.3 was considered negative according to the manufacturer's instructions.

Heparin induced platelet activation (HIPA) assay

All analyses were performed on fresh, unfrozen blood samples. Upon arrival at the laboratory, samples were immediately centrifuged to obtain serum. Prior to testing, the sera samples were heat inactivated at 56 ◦C for 30 minutes. The HIPA assay was performed within three days of sample receipt, as previously described.[1] All HIPA assays were performed by experienced laboratory technicians according to standardized operating procedures with internal quality controls. HIPA results were interpreted by a laboratory physician with expertise in platelet immunology in collaboration with the performing technician. Neither the technician nor the laboratory physician was blinded to clinical data and immunoassay results. A HIPA assay was considered positive if platelet activation was observed in at least two out of four donor platelet suspensions in the presence of low heparin concentration (0.2 IU/mL), and a negative reaction occurred in the presence of high heparin concentration (100 IU/mL).

Diagnostic algorithm for HIT diagnosis

Clinical probability of HIT was determined by the 4Ts score.[2] All samples underwent both immunoassays (CLIA and ELISA) as well as the functional assay (HIPA), irrespective of the 4Ts score. HIT diagnosis was based on a positive HIPA result. In cases with indetermined HIPA but a strongly positive ELISA (OD >1.0), clinical compatibility was assessed by two experienced physicians who had

access to all clinical and laboratory data including immunoassay results. Clinical compatibility was defined as platelet count changes without alternative explanations, concomitant D-dimer increase, platelet count recovery after initiation of alternative anticoagulation, or a positive functional assay on repeat testing. HIT was excluded if HIPA was negative and ELISA was negative or weakly positive (OD <1.0). The outcome assessment methodology was applied uniformly across all patients regardless of demographic characteristics, clinical specialty, or treatment modality.

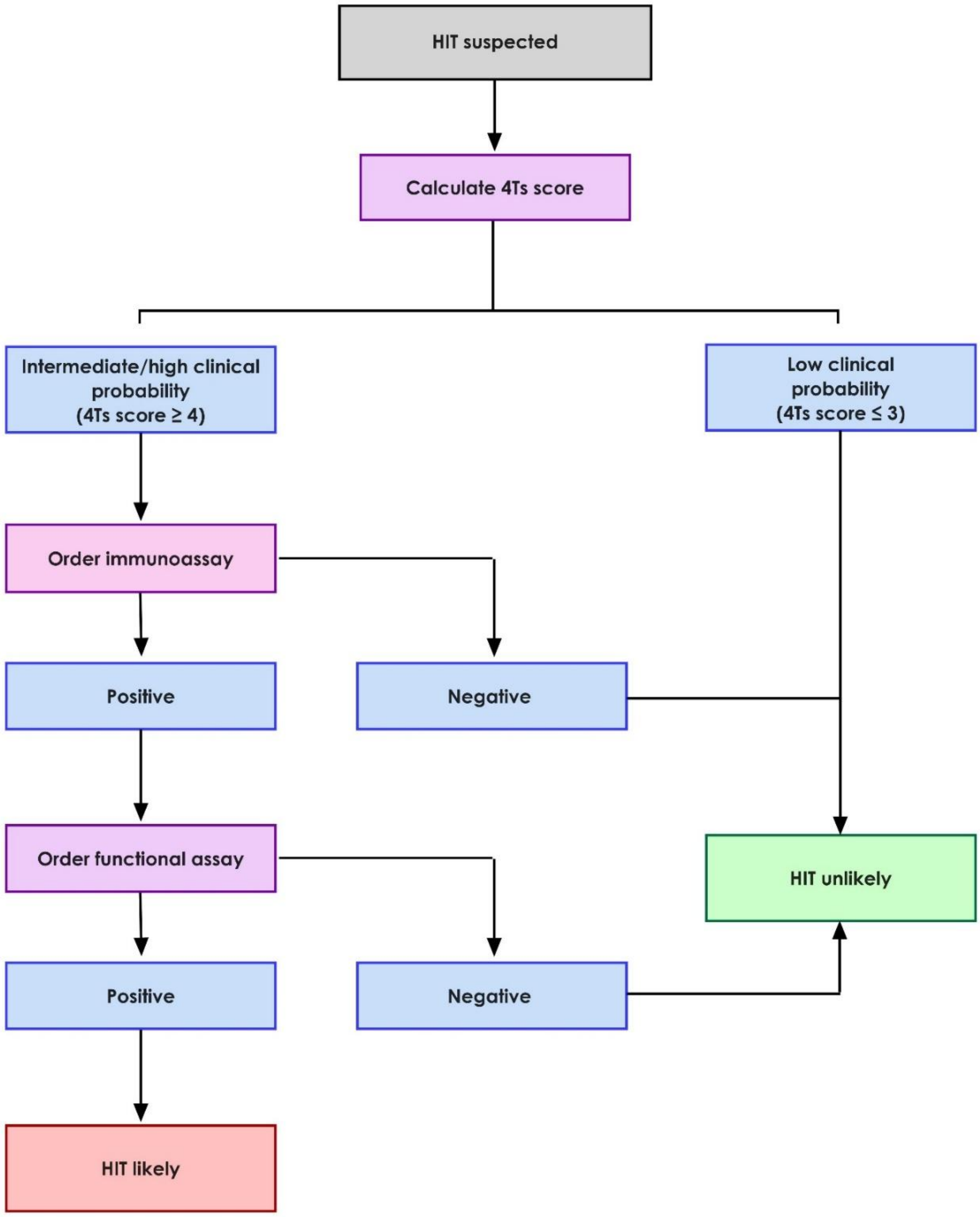

Supplemental Figure 1. Diagnostic algorithm for heparin-induced thrombocytopenia (HIT) based on 2018 ASH guideline.[2]

ASH recommended diagnostic algorithm for HIT diagnosis

We assessed the diagnostic performance of the American Society of Hematology (ASH) guideline–recommended algorithm (Figure S1) for suspected HIT in our patient cohort.[2] According to the 2018 ASH guidelines, the workup begins with clinical probability assessment using the 4Ts score.[2] Patients with a low 4Ts score (≤3) are considered unlikely to have HIT and laboratory testing is not recommended. Patients with an intermediate or high 4Ts score (≥4) should undergo immunoassay testing for anti-PF4/heparin antibodies. A negative immunoassay excludes HIT, whereas a positive immunoassay warrants confirmatory testing with a functional platelet activation assay, such as the serotonin release assay (SRA) or HIPA assay, when available. A positive functional assay establishes the diagnosis of HIT, while a negative functional assay generally rules it out. However, in patients with a high clinical probability and a strongly positive immunoassay, HIT may still be considered despite a negative functional assay, given the possibility of false-negative results.

Development of the TuHIT score

Predictor selection and definition

The predictors included in the TuHIT score were selected a priori based on their established role in HIT diagnosis as recommended by the 2018 AS H guideline and their inclusion in existing prediction models and diagnostic algorithms.[3-5] Specifically, we selected: (1) the 4Ts clinical probability score, (2) CLIA, and (3) ELISA. These three components represent the core elements of the ASH-recommended diagnostic pathway and have been incorporated into several published HIT prediction models, including the Lausanne Bayesian algorithms, the Hamilton algorithm, and the TORADI-HIT score. No additional predictors were considered to maintain clinical simplicity and ensure the score could be implemented using readily available diagnostic tools in most clinical laboratories.

The 4Ts score is a clinical probability assessment tool that evaluates four key features: magnitude of platelet count fall, timing of platelet count fall, presence of thrombosis or other sequelae, and likelihood of other causes of thrombocytopenia. 4Ts scores range from 0 to 8 points and were initially calculated by treating physicians at the time of sample submission using standardized forms. For the purposes of this study, all 4Ts scores were independently verified and recalculated by two experienced physicians through systematic review of clinical patient records to ensure accuracy and consistency. CLIA and ELISA were performed according to manufacturer recommendations with internal quality controls, with values measured at the time of diagnostic testing. Multiple clinically relevant cut-offs were used to stratify immunoassay results into categories for point assignment (see Analytical Methods for detailed cut-off definitions and rationale).

Sample size calculation

No formal a priori sample size calculation was performed. The study included all consecutive patients referred with suspected HIT during the study period. Methodological approaches to address the small number of events are described in the Statistical Analysis section. There were no missing data for the primary variables required for TuHIT score calculation (4Ts score, CLIA results, ELISA results) and HIPA outcomes.

Logistic regression and internal validation

All 343 patients with complete predictor and outcome data were used for model development. No data partitioning was performed. Instead, model stability and optimism were assessed through bootstrap internal validation as described below.

The 4Ts score was categorized according to established ASH guideline thresholds: low (0-3 points), intermediate (4-5 points), and high (6-8 points).

CLIA results were categorized using following thresholds.

0.13 U/mL : based on published literature as a rule-out cut-off.[6]
0.55 U/mL : optimal cut-off based on ROC analysis (Figure 1D) in the current study.
1.0 U/mL : manufacturer recommended threshold (HemosIL AcuStar HIT-IgG(PF4-H) assay).
3.0 U/mL : based on published literature as a rule-in cut-off.[6]

For ELISA, we used following thresholds to define the categories:

ELISA ≥0.3 OD : manufacturer recommended negative threshold (Zymutest HIA IgG ELISA Kit)
ELISA 0.6 OD : optimal cut-off based on ROC analysis (Figure 1E) in the current study.
ELISA 1.0 OD : based on published literature.[7]
ELISA 1.5 OD : threshold for high/very high probability based on published literature.[7,8]

Based on these cut-offs, we grouped CLIA results and ELISA results into 5 subgroups (Table S1). This multi-threshold categorization strategy integrates evidence from published literature, manufacturer recommendations, and data-driven optimization in our cohort.

Supplemental Table 1. Predictors and point scores

Parameter	Value	Points
4T Score	Low	0
	Intermediate	2
	High	4
CLIA	< 0.13 U/mL	0
	0.13 to <0.55 U/mL	1

	0.55 to <1.0 U/mL	2
	1.0 to <3.0 U/mL	3
	≥3.0 U/mL	4
ELISA	< 0.3 OD	0
	0.3 to <0.6 OD	3
	0.6 to <1.0 OD	6
	1.0 to <1.5 OD	9
	≥1.5 OD	12

The TuHIT score was developed using Firth's penalized maximum likelihood logistic regression.[9] Complete regression output including coefficients, standard errors, odds ratios, and confidence intervals is provided in Supplemental Table 2.

Supplemental Table 2. Multivariable analysis using Firth's penalized maximum likelihood estimation

Variable	Coefficient (β)	SE	95% CI		OR	95% CI		*p* value
			Lower	Upper		Lower	Upper	
Intercept	-10.69	2.11	-16.51	-7.09	—	—	—	<0.001
4Ts Score	1.04	0.69	-0.37	2.66	2.83	0.69	14.22	0.150
CLIA	0.59	0.28	0.01	1.21	1.81	1.01	3.34	0.045
ELISA	1.64	0.34	1.01	2.53	5.17	2.76	12.50	<0.001

Abbreviations: SE, standard error; CI, confidence interval; OR, odds ratio; CLIA, chemiluminescence immunoassay; ELISA, enzyme-linked immunosorbent assay; OD, optical density.

Point values were derived directly from regression coefficients to maintain proportionality to predictor strength:

First, we extracted the regression coefficients from Firth's model

- β(4Ts) = 1.039, β(CLIA) = 0.592, β(ELISA) = 1.642

All coefficients were normalized by dividing each with the smallest coefficient (|β|_min = 0.592). Approximate points were assigned to each parameter.

- 4Ts: 1.04 ÷ 0.59 = 1.75 ≈ 2 points per category
- CLIA: 0.59 ÷ 0.59 = 1.00 ≈ 1 point per category
- ELISA: 1.64 ÷ 0.59 = 2.77 ≈ 3 points per category

Points for each category level were calculated as (category number - 1) × points per category, creating an integer-based additive score ranging from 0 to 20 points. The detailed point allocation is shown in Table S2.

Study registration:

No formal study protocol was prospectively registered or published. This was a retrospective diagnostic accuracy study using existing clinical data.

Data availability:

Due to privacy regulations (GDPR) and institutional policies, individual patient data cannot be made publicly available. Anonymized data may be available from the corresponding author upon reasonable request, subject to approval by the institutional ethics committee and establishment of a data sharing agreement. Requests should specify the intended use, analysis plan, and data protection measures.

Code availability:

The R code used for Firth's penalized logistic regression, bootstrap validation, and ROC analysis is available from the corresponding author upon reasonable request. The code includes scripts for model development, point assignment calculation, and performance evaluation. All analyses used publicly available R packages as specified in the Methods section.

Patient and public involvement:

No patients or members of the public were involved in the design, conduct, or reporting of this study.

Statistics

Statistical analyses were performed using GraphPad Prism (version 10.3.0, GraphPad Software, San Diego, CA, USA) and R version 4.5.1 (R Foundation for Statistical Computing, Vienna, Austria). Firth's penalized logistic regression and bootstrap validation were implemented in R using the logistf (v1.26.1) and boot (v1.3-30) packages, respectively. Intergroup comparisons were performed using the Mann–Whitney U test for continuous variables and Fisher's exact test for categorical variables. Receiver-operating characteristic (ROC) curve analysis was performed using GraphPad Prism to determine optimal cut-off values for in predicting HIT. The optimal cut-off for each assay (4Ts score, immunoassays, and TuHIT score) was determined using Youden's Index. Diagnostic performance was evaluated by calculating sensitivity, specificity, positive predictive value (PPV), and negative predictive value (NPV). A p-value <0.05 was considered statistically significant in all tests.

Results

Study cohort

After excluding 57 repeat samples, 343 patients were included (Figure S2). The cohort comprised 215 males (62.7%) and 128 females (37.3%) with a median age of 65 years (IQR: 54-74). There were no missing data for the main parameters (4Ts, CLIA, ELISA, HIPA). Twenty patients (5.8%) were diagnosed with HIT (Table S3).

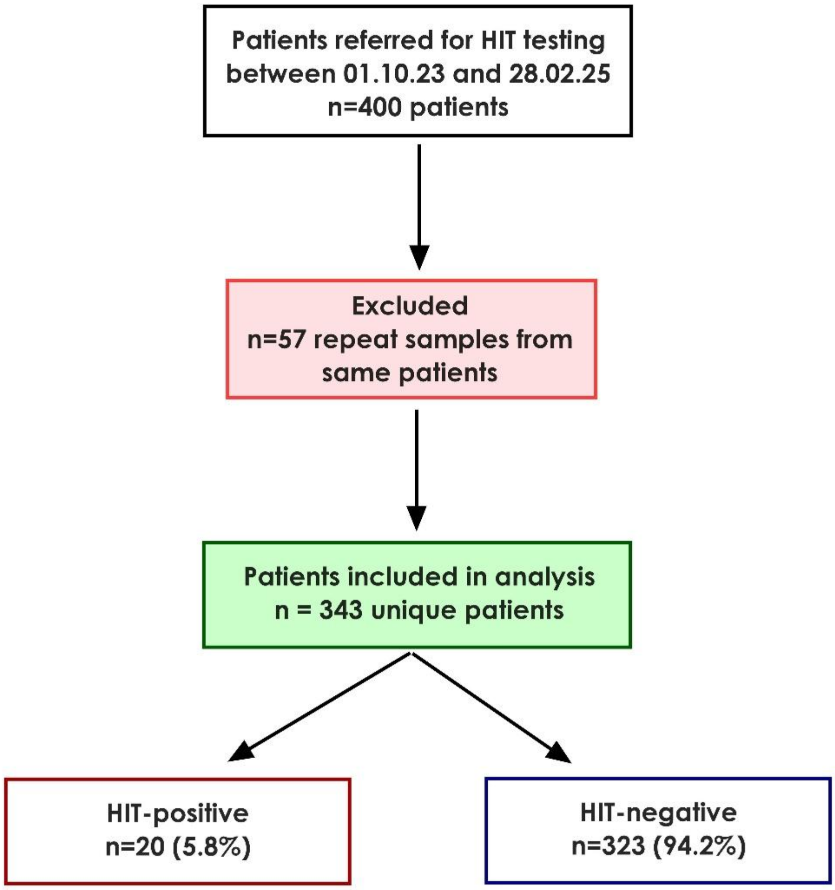

Supplemental Figure 2. Patient flow diagram. Flowchart of patient selection. Of 400 samples received for HIT testing, 57 repeat samples were excluded, yielding 343 unique patients for analysis. Final diagnosis: 20 HIT-positive (5.8%), 323 HIT-negative (94.2%).

The study cohort was representative of the typical HIT referral population at our institution, including patients from diverse clinical specialties (medical, surgical, intensive care) without exclusion based on demographic characteristics such as age, sex, or underlying disease. Subgroup analyses by demographic characteristics were not performed due to the small number of HIT cases.

Supplemental Table 3. Patient Characteristics

Characteristic	**All patients (n 343)**	**HIT Positive (n 20)**	**HIT Negative (n 323)**
Age, median (IQR), y	65 (55-74)	63 (43-71)	65 (55-74)
Sex, n (%)			
Female	128 (37.3)	6 (30.0)	122 (37.8)
Male	215 (62.7)	14 (70.0)	201 (62.2)
Clinical setting, n (%)			
Internal medicine	118 (34.4)	6 (30.0)	112 (34.7)
Cardiovascular surgery	98 (28.6)	5 (25.0)	93 (28.8)
Major trauma & orthopedic surgery	23 (6.7)	3 (15.0)	20 (6.2)
Other surgery	85 (24.8)	2 (10)	83 (25.7)
ECLS	19 (5.5)	4 (20)	15 (4.6)
Platelet count, median (IQR), 10^9/L	76 (54-103)	91 (50-119)	75 (54-102)
CLIA, median (IQR), U/ml	0.08 (0.04-0.20)	6.35 (1.95-11.51)	0.07 (0.04-0.18)
ELISA, median (IQR), OD	0.15 (0.11-0.26)	2.24 (1.37-2.71)	0.14 (0.10-0.23)
4Ts score, n (%)			
Low risk (0-3)	133 (38.8)	1(5.0)	132 (40.9)
Intermediate risk (4-5)	166 (48.4)	11 (55.0)	155 (48.0)
High risk (6-8)	44 (12.8)	8 (40)	36 (11.1)
CLIA, n (%), U/ml			
<0.13	217 (63.3)	1 (5.0)	216 (66.9)
13 to <0.55	81 (23.6)	1 (5.0)	80 (24.8)
0.55 to <1.0	13 (3.8)	2 (10.0)	11 (3.4)
1.0 to <3.0	14 (4.1)	4 (20.0)	10 (3.1)
≥3.0	18 (5.2)	12 (60.0)	6 (1.9)
ELISA, n(%), OD			
<0.3	267 (77.8)	0 (0.0)	267 (82.7)
0.3 to <0.6	39 (11.4)	2 (10.0)	37 (11.5)
0.6 to <1.0	14 (4.1)	1 (5.0)	13 (4.0)
1.0 to <1.5	9 (2.6)	3 (15.0)	6 (1.9)
≥1.5	14 (4.1)	14 (70.0)	0 (0.0)

Model performance evaluation

The TuHIT score produces an additive point-based risk score ranging from 0 to 20 points. Based on ROC analysis, a threshold of ≥10 points was established as the diagnostic cut-off for HIT, optimized to maximize sensitivity of 95% (75.1–99.8), while maintaining specificity at 97.2% (94.8–98.7)). Patients with scores ≥10 are classified as high risk for HIT, warranting either immediate alternative anticoagulation or confirmatory functional testing depending on clinical context, while patients with scores <10 are classified as low risk, where HIT can be safely excluded without further testing.

Bootstrap internal validation

Bootstrap internal validation with 1,000 resamples was performed to assess model stability and quantify optimism in model performance using nonparametric bootstrap with replacement (random seed = 123 for reproducibility). Bootstrap validation demonstrated exceptional model stability. The original AUC of 0.9926 (95% CI: 0.9823-0.9991) showed negligible optimism (0.0000, SE: 0.0046), yielding an optimism-corrected AUC of 0.9926. The minimal optimism indicates virtually no overfitting despite the small sample size.

References

1. Greinacher A, Michels I, Kiefel V, Mueller-Eckhardt C. A rapid and sensitive test for diagnosing heparin-associated thrombocytopenia. *Thromb Haemost*. Dec 2 1991;66(6):734-6.
2. Cuker A, Arepally GM, Chong BH, et al. American Society of Hematology 2018 guidelines for management of venous thromboembolism: heparin-induced thrombocytopenia. *Blood Adv*. Nov 27 2018;2(22):3360-3392. doi:10.1182/bloodadvances.2018024489
3. Larsen EL, Nilius H, Studt JD, et al. Accuracy of Diagnosing Heparin-Induced Thrombocytopenia. *JAMA Netw Open*. Mar 4 2024;7(3):e243786. doi:10.1001/jamanetworkopen.2024.3786
4. Nilius H, Cuker A, Haug S, et al. A machine-learning model for reducing misdiagnosis in heparin-induced thrombocytopenia: A prospective, multicenter, observational study. *EClinicalMedicine*. Jan 2023;55:101745. doi:10.1016/j.eclinm.2022.101745
5. Bakchoul T, Giptner A, Najaoui A, Bein G, Santoso S, Sachs UJ. Prospective evaluation of PF4/heparin immunoassays for the diagnosis of heparin-induced thrombocytopenia. *J Thromb Haemost*. Aug 2009;7(8):1260-5. doi:10.1111/j.1538-7836.2009.03465.x
6. Steinauer T, Matthey-Guirao E, Gomez FJ, et al. Sequential combinations of rapid immunoassays for prompt recognition of heparin-induced thrombocytopenia. *Blood*. Aug 14 2025;146(7):887-896. doi:10.1182/blood.2024027517
7. Nellen V, Sulzer I, Barizzi G, Lammle B, Alberio L. Rapid exclusion or confirmation of heparin-induced thrombocytopenia: a single-center experience with 1,291 patients. *Haematologica*. Jan 2012;97(1):89-97. doi:10.3324/haematol.2011.048074
8. Raschke RA, Gallo T, Curry SC, et al. Clinical effectiveness of a Bayesian algorithm for the diagnosis and management of heparin-induced thrombocytopenia. *J Thromb Haemost*. Aug 2017;15(8):1640-1645. doi:10.1111/jth.13758
9. Firth D. Bias Reduction of Maximum-Likelihood-Estimates. *Biometrika*. Mar 1993;80(1):27-38. doi:Doi 10.2307/2336755
